# Long-term Outcomes After Anterior Cruciate Ligament Reconstruction With 3 Different Surgical Techniques: A Prospective Randomized Clinical and Radiographic Evaluation at a Minimum of 20 Years’ Follow-up

**DOI:** 10.1177/23259671241302348

**Published:** 2025-01-29

**Authors:** Gian Andrea Lucidi, Piero Agostinone, Stefano Di Paolo, Giacomo Dal Fabbro, Margherita Serra, Marianna Viotto, Alberto Grassi, Stefano Zaffagnini

**Affiliations:** * Clinica Ortopedica e Traumatologica II, Istituto Ortopedico Rizzoli, Bologna, Italy; † Department of Biomedical and Neuromotor Sciences (DIBINEM), University of Bologna, Bologna, Italy; Investigation performed at Clinica Ortopedica e Traumatologica II, Istituto Ortopedico Rizzoli, Bologna, Italy

**Keywords:** knee ligaments, ACL, lateral extra-articular tenodesis, bone–patellar tendon–bone, hamstring tendon, long-term follow-up

## Abstract

**Background:**

In recent years, lateral extra-articular tenodesis (LET) has been shown to be promising in reducing the graft failure rate at short-term follow-up. However, there is a lack of studies investigating the incidence of complications and lateral osteoarthritis (OA) after this procedure, and only a few studies have reported long-term results after anterior cruciate ligament (ACL) reconstruction.

**Purpose/Hypothesis:**

This study aimed to compare the failure rate, clinical outcomes, and OA incidence of 3 different ACL reconstruction techniques: single-bundle quadrupled hamstring tendon (HT), bone–patellar tendon–bone (BPTB), and over-the-top HT plus LET (HT + LET). The authors hypothesized that the 3 techniques would have comparable clinical and radiographic outcomes at long-term follow-up.

**Study Design:**

Randomized controlled trial; Level of evidence, 1.

**Methods:**

A total of 75 patients were included in this prospective study and randomized to undergo 1 of 3 ACL reconstruction techniques. At the last follow-up (minimum of 20 years), patient-reported outcome measure (PROM) scores, complications, and reoperations were collected, and an objective clinical evaluation was performed, including the measurement of anteroposterior (AP) laxity using an arthrometer and the quantification of the pivot shift (PS) using a triaxial accelerometer. Clinical failure was considered in patients with evidence of a graft rupture or those with a side-to-side difference in AP laxity >5 mm or with a side-to-side difference in the PS >1.5 mm/s^2^. At the last follow-up, patients also underwent a radiographic evaluation to assess the incidence of tibiofemoral and patellofemoral OA.

**Results:**

PROM scores were collected from 61 patients (81%) at a mean follow-up of 23.0 ± 1.1 years. Of the 75 patients, 37 (49%) completed the clinical evaluation, and 35 (47%) had radiographs obtained. Regarding the PROMs, the HT + LET group showed a slightly higher Tegner score than the BPTB group (*P* = .023). All other PROM scores were not significantly different between groups. The revision and clinical failure rates were 16% and 37%, respectively, for the BPTB group, 10% and 25%, respectively, for the HT group, and 5% and 19%, respectively, for the HT + LET group, with no statistical difference between the groups. The side-to-side difference in AP laxity was lower in the BPTB group than in the HT group (*P* = .049). The BPTB group showed a higher patellofemoral OA rate than the HT + LET group (*P* = .029). There was no difference in the incidence of lateral OA between the 3 techniques.

**Conclusion:**

The 3 different surgical techniques achieved satisfactory clinical outcomes after ACL reconstruction at long-term follow-up. However, the BPTB group was associated with an increased incidence of patellofemoral OA. Also, the HT + LET group was associated with a slightly increased Tegner score at long-term follow-up, but there was no evidence of an increased risk of lateral OA for the HT + LET group.

Anterior cruciate ligament (ACL) tears are one of the most frequent injuries affecting the knee joint in young and active patients.^11,12,21,32^ Regardless of the advancement in knee anatomy, biomechanics, and surgical techniques, graft choice remains one of the most controversial and investigated topics in modern ACL literature. The autologous hamstring tendon (HT) and bone–patellar tendon–bone (BPTB) are the most used grafts for ACL reconstruction,^1,22,24,28^ and each of them has advantages and disadvantages. The patellar tendon has been the historical gold standard, and several surgeons prefer it, especially in athletes,^11,24,25^ because of its high ultimate load to failure,^24,31^ its faster integration during the bone healing process,^22,25,35^ and its supposedly lower rate of failure compared to the HT.^1,6,22,25,35^ On the other hand, the HT is associated with a lower incidence of donor site morbidity, no risk of patellar fractures, and reduced extensor mechanism damage.^6,28,35^

In recent years, there has been an increased interest in the association of lateral extra-articular tenodesis (LET) with intra-articular ACL reconstruction to improve rotational instability.^13,17,19,23^ Recent studies have shown that LET could reduce the graft failure rate in a high-risk population.^8,17,19,23^ Because of increasing evidence, previous literature has recommended the use of LET in addition to ACL reconstruction in high-demand patients and revision surgery.^7^ However, LET is historically considered to be at risk of lateral compartment overconstraint, which could increase the incidence of lateral knee osteoarthritis (OA). Meanwhile, recent literature has revealed conflicting biomechanical and clinical findings.^2,4,29,30^

It is difficult to evaluate the risk of OA onset related to different ACL surgical techniques because only a few studies are available with an appropriate design and follow-up time.^11^ In a comparative study with 20 years of follow-up, Castoldi et al^2^ found an increased risk of lateral OA in patients who underwent ACL reconstruction with LET. However, the high incidence of lateral meniscectomy in the 2 investigated groups made this result unreliable. Another study by the SANTI Study Group^30^ found an increased risk of medial OA in patients who underwent ACL reconstruction with the patellar tendon compared to ACL reconstruction with the HT and LET at a mean follow-up of 104 months. Additionally, the association of BPTB grafts and patellofemoral OA is still controversial.^14,26,32^

The aim of the present study was to compare 3 different ACL reconstruction techniques in terms of the failure rate, clinical outcomes, and OA incidence including single-bundle quadrupled HT, BPTB, and over-the-top HT plus LET (HT + LET) at a minimum of 20 years’ follow-up. Our primary hypothesis was that the HT + LET group would not be inferior regarding clinical scores, the failure rate, and knee laxity. The secondary hypothesis was that the HT + LET group would not have an increased risk of lateral OA, while the BPTB group would be associated with a higher incidence of patellofemoral OA.

## Methods

This study was approved by the institutional review board of Istituto Ortopedico Rizzoli (No. 112/98). All the patients signed an informed consent form.

### Patient Selection and Randomization

A prospective randomized study was designed to compare 3 different techniques of ACL reconstruction in 1998.^35^ A single surgeon (S.Z.) performed all the surgical procedures. The inclusion criteria included the following: no prior knee surgery, no meniscal injury, a healthy contralateral knee, no chondral injury, no sign of joint degenerative changes, no history of patellofemoral pain, age <50 years, an intact posterior cruciate ligament, and a maximum grade 1 medial collateral ligament injury. Before the injury, all patients were involved in cutting sports at a competitive or amateur level. Randomization was conducted using sealed opaque envelopes to divide patients into 3 groups: The BPTB group underwent ACL reconstruction with a BPTB graft, the HT group underwent ACL reconstruction with a 4-strand HT graft, and the HT + LET group underwent over-the-top ACL reconstruction with an HT graft plus LET. The patients were evaluated at a minimum follow-up of 20 years. The present study reports data acquired at the final evaluation.

### Surgical Techniques

#### BPTB Group

**Figure 1.**
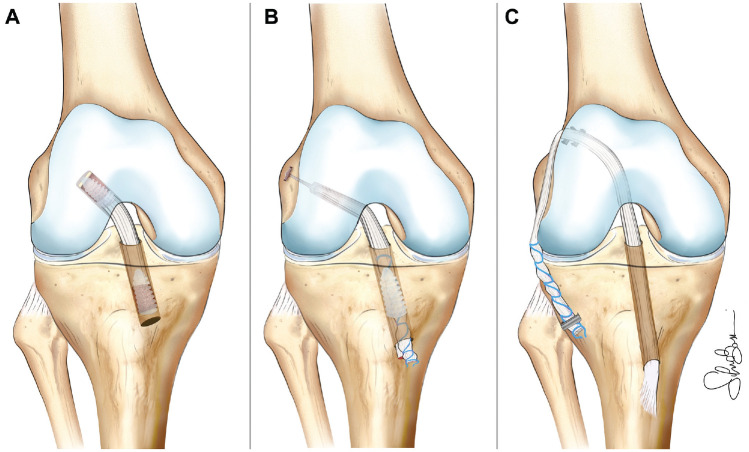
Representation of the 3 surgical techniques investigated: (A) anatomic anterior cruciate ligament (ACL) reconstruction with a bone–patellar tendon–bone, (B) anatomic single-bundle ACL reconstruction with a quadrupled hamstring tendon, and (C) over-the-top ACL reconstruction with a hamstring tendon plus lateral extra-articular tenodesis.

A midline skin incision was made on the patellar tendon, and the third central portion of the tendon was harvested with bone plugs (~25 mm in length) taken from the patella and the tibial tubercle. The diameter of the graft harvested was approximately 9 mm. A 2-mm pin was placed to guide subsequent tibial tunnel drilling; the exit point into the joint was at the junction of the middle and posterior thirds of the native ACL footprint. The femoral half-tunnel was created using the transtibial technique, and the depth was approximately 3 cm. Then, the graft was passed through the tibial and femoral tunnels and fixed at the femur with a metal interference screw (Smith & Nephew). Overall, 10 cycles of knee flexion-extension were performed for pretensioning, and tibial fixation was conducted at 20° of knee flexion with another interference screw (Figure 1A).

#### HT Group

A skin incision was made over the pes anserinus. Semitendinosus and gracilis tendons were harvested using a tendon stripper and detached by their tibial insertion; the graft was prepared to obtain a 4-strand construct with a diameter of 8 to 9 mm. Tibial and femoral tunnels were created as previously described for the BPTB group. The graft was passed through the tibial tunnel into the joint and subsequently into the femoral half-tunnel. Femoral fixation was achieved with a suspensory fixation device (Smith & Nephew), and tibial fixation was performed with an absorbable interference screw (Smith & Nephew) with the knee flexed at 20° (Figure 1B).

#### HT + LET Group

This group of patients underwent over-the-top ACL reconstruction with an HT graft plus LET.^34^ A skin incision on the pes anserinus exposed the gracilis and semitendinosus tendons, which were harvested proximally with a tendon stripper. The tibial insertion was left intact, and the 2 tendons were sutured together to obtain a double-strand construct (~7-8 mm in diameter). The tibial tunnel was drilled in the direction of the posteromedial portion of the native ACL footprint. The graft was then passed into the tibial tunnel and the joint to reach the over-the-top position of the lateral femoral condyle, where it was fixed with 2 metal staples (Citieffe) with the knee at 90° of flexion. The remaining part of the graft was passed below the iliotibial band but superficial to the lateral collateral ligament and pulled distally to the tibia, where it was fixed at the level of the Gerdy tubercle with a metal staple (Figure 1C).

### Rehabilitation

The 3 groups underwent the same postoperative rehabilitation protocol. On the day after surgery, isometric quadriceps strengthening and hamstring stretching exercises were allowed. Passive flexion and extension were allowed from the third postoperative day. Patients were permitted to walk with the help of crutches and no knee bracing, with partial weightbearing for the first 2 weeks and then complete weightbearing from the third week. Stationary bicycles, knee active extension, and one-quarter squats were introduced in the fourth week. Straight running was allowed after 3 months and sports activities after 5 months. Complete return to sports was decided utilizing a multidisciplinary approach, considering the differences in muscle atrophy and the single-leg hop between the operated and contralateral legs. This typically occurred at least 6 months after surgery, in line with our standard protocol at that time.

### Data Collection

After the acquisition of informed consent at a minimum follow-up of 20 years, every patient completed a self-administrated questionnaire including the visual analog scale for pain, the Western Ontario and McMaster Universities Arthritis Index, the International Knee Documentation Committee (IKDC) form, the Tegner activity scale, the Lysholm knee scoring scale, and the Knee injury and Osteoarthritis Outcome Score. A clinical examination of the knee was performed according to the objective IKDC form; the thigh circumference 5 and 15 cm above the patella was also measured for both limbs. Knee laxity was assessed using an arthrometer (KT-1000 arthrometer; Genourob) for anteroposterior (AP) laxity and a triaxial accelerometer (KiRA; Orthokey) for pivot shift (PS). The same orthopaedic surgeon performed all clinical evaluations (G.A.L.). Information about anterior knee pain and subsequent surgery was collected. Clinical failure was considered in patients with evidence of a graft rupture or those with a side-to-side difference in AP laxity >5 mm or with a side-to-side difference in the PS >1.5 mm/s^2^ (objective IKDC grade C-D).^3,10,20^ Patients who underwent ACL revision were excluded from the clinical examination.

**Figure 2.**
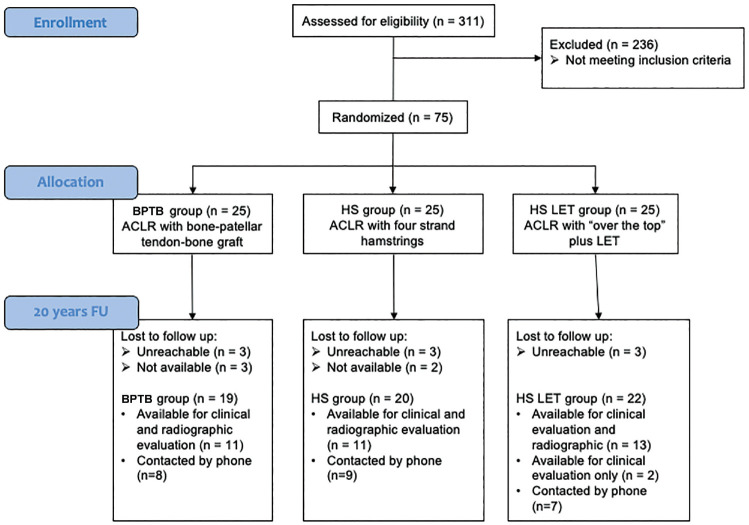
CONSORT (Consolidated Standards of Reporting Trials) flow diagram of patient inclusion and exclusion criteria. ACLR, anterior cruciate ligament reconstruction; BPTB, bone–patellar tendon–bone; FU, follow-up; HS, hamstring tendon; LET, lateral extra-articular tenodesis; phone, telephone.

Knee radiographs in the Rosenberg view and axial projections were acquired from each patient.^16^ The Kellgren-Lawrence classification^16^ was used to evaluate tibiofemoral OA. The Iwano classification^15^ was used to examine patellofemoral OA. Radiographs were evaluated by 2 orthopaedic surgeons (P.A. and A.G.), and when they disagreed, a third senior surgeon (S.Z.) made the final decision. Patients not available for the in-office clinical and radiographic examinations were contacted by telephone (Figure 2).

### Statistical Analysis

The normal distribution of data was verified through the Shapiro-Wilk test. Normally distributed continuous variables were presented as the mean and standard deviation; the Tegner score was presented as the median and range. Categorical variables were presented as numbers and percentages. To assess the between-group difference among the 3 surgical techniques over time for continuous variables, 2-way repeated-measures analysis of variance was performed. A priori power analysis was conducted to determine an adequate sample size. Analysis of variance (*F* test) was used for the comparison of the 3 surgical techniques. Considering a difference between groups of 8 points for the Lysholm score and a standard deviation within groups of 10 points, a minimum of 20 participants per group would be required to have a power of 0.8 with an alpha of .05.^18^

For multiple comparisons, the 2-tailed Student *t* test was used, and *P* values were adjusted using the Bonferroni post hoc correction. The chi-square test was performed to assess the differences in categorical variables. Differences between the groups were considered statistically significant if *P* < .05. Statistical analysis was performed using SPSS software (Version 26.0; IBM).

## Results

Of 311 patients who underwent ACL reconstruction during the enrollment period, 75 patients met the inclusion criteria and were included in this study. The 75 patients were randomized into 3 groups, with 25 each; all were available for the intermediate evaluation. At the final follow-up, 61 patients (81%) completed the questionnaire of patient-reported outcome measures (PROMs) at a mean follow-up of 23.0 ± 1.1 years. Of these, 24 patients were unavailable for a clinical examination and were contacted by telephone. Therefore, 37 patients (49%) underwent the clinical examination, while only 35 underwent knee radiography (2 patients in the HT + LET group declined their consent for radiation exposure) (Figure 2).

The characteristics of the patients are reported in Table 1. A significant difference between the groups was found only for body mass index, which was higher in the BPTB group compared to the HT and HT + LET groups at the final follow-up.

**Table 1:** Patient Characteristics at Baseline and Final Follow-up^*a*^

	BPTB Group (n = 19)	HT Group (n = 20)	HT + LET Group (n = 22)	*P*
Sex, male/female	13 (68)/6 (32)	13 (65)/7 (35)	15 (68)/7 (32)	NS
Knee, right/left	12 (63)/7 (37)	11 (55)/9 (45)	9 (41)/13 (59)	NS
Age at surgery, y	29.3 ± 7.2	31.3 ± 7.6	27.9 ± 7.1	NS
Age at final follow-up, y	52.8 ± 7.3	54.5 ± 7.5	51.3 ± 7.4	NS
Follow-up, y	23.1 ± 1.1	22.9 ± 1.2	23.0 ± 1.1	NS
BMI at surgery, kg/m^2^	25.0 ± 3.3	24.0 ± 3.7	23.0 ± 2.8	NS
BMI at final follow-up, kg/m^2^	26.4 ± 2.7	24.3 ± 3.2	24.9 ± 2.9	.024

a Data are shown as n (%) or mean ± SD. The final follow-up was 23.0 ± 1.1 years. BMI, body mass index; BPTB, bone–patellar tendon–bone; HT, hamstring tendon; HT + LET, hamstring tendon plus lateral extra-articular tenodesis; NS, not significant.

### PROM Scores and Clinical Findings

**Figure 3.**
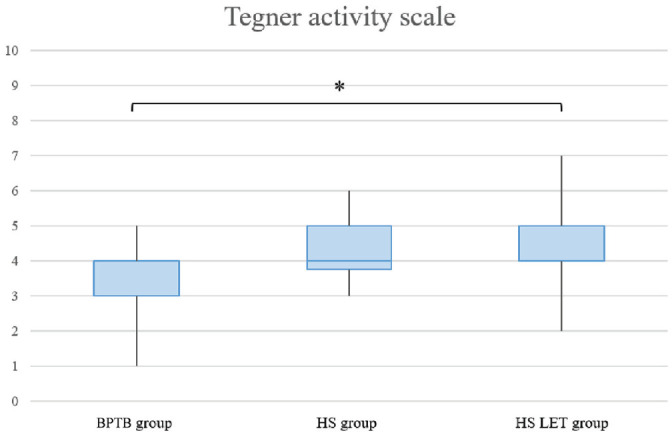
Graphical representation of the Tegner score. The asterisk indicates a statistically significant difference between the bone–patellar tendon–bone (BPTB) group and the hamstring tendon plus lateral extra-articular tenodesis (HS LET) group. HS, hamstring tendon.

Analysis of PROMs revealed no differences between the 3 surgical groups, except for the Tegner score, which was significantly greater in the HT + LET group compared with the BPTB group (*P* = .023) (Table 2 and Figure 3).

**Table 2:** PROM Scores at Final Follow-up^*a*^

	BPTB Group (n = 19)	HT Group (n = 20)	HT + LET Group (n = 22)
Tegner activity scale, median (range)	3 (1-5)	4 (3-6)	4 (2-7)^*b*^
Lysholm knee scoring scale	84.8 ± 10.3	88.6 ± 8.3	87.0 ± 9.5
VAS pain	2.6 ± 2.2	2.7 ± 1.8	2.2 ± 2.0
KOOS Symptoms	80.4 ± 11.2	82.1 ± 12.3	86.6 ± 11.8
KOOS Pain	86.4 ± 11.2	90.7 ± 6.6	90.0 ± 10.3
KOOS Activities of Daily Living	92.0 ± 10.0	95.7 ± 3.8	94.5 ± 8.9
KOOS Sports	72.2 ± 18.2	81.0 ± 12.1	78.4 ± 21.3
KOOS Quality of Life	80.2 ± 14.1	87.5 ± 9.3	86.3 ± 14.3
Subjective IKDC form	78.4 ± 13.7	81.6 ± 11.9	81.0 ± 14.5
WOMAC	88.5 ± 9.5	91.6 ± 5.9	92.0 ± 9.2

a Data are shown as mean ± SD unless otherwise indicated. The final follow-up was 23.0 ± 1.1 years. BPTB, bone–patellar tendon–bone; HT, hamstring tendon; HT + LET, hamstring tendon plus lateral extra-articular tenodesis; IKDC, International Knee Documentation Committee; KOOS, Knee injury and Osteoarthritis Outcome Score; PROM, patient-reported outcome measure; VAS, visual analog scale; WOMAC, Western Ontario and McMaster Universities Arthritis Index.

b Statistically significant difference compared with the BPTB group (*P* = .023).

After excluding patients who underwent ACL revision, no statistically significant difference was found between the groups for the objective IKDC grade. Most of the patients were grade A or B: 73% in the BPTB group, 82% in the HT group, and 94% in the HT + LET group (*P* > .05) (Table 3). The side-to-side difference in thigh circumference 5 and 15 cm above the patella was similar among the groups, and no statistically significant difference was found (*P* > .05) (Table 4).

**Table 3:** Objective IKDC Grades^*a*^

Grade	BPTB Group (n = 11)	HT Group (n = 11)	HT + LET Group (n = 15)
A	1 (9)	0 (0)	4 (27)
B	7 (64)	9 (82)	10 (67)
C	2 (18)	2 (18)	1 (6)
D	1 (9)	0 (0)	0 (0)

a Data are shown as n (%). BPTB, bone–patellar tendon–bone; HT, hamstring tendon; HT + LET, hamstring tendon plus lateral extra-articular tenodesis; IKDC, International Knee Documentation Committee.

**Table 4:** Side-to-Side Differences in Thigh Circumference and Knee Laxity^*a*^

	BPTB Group	HT Group	HT + LET Group
Thigh circumference 5 cm above patella, cm	0.4 ± 1.5	0.5 ± 0.8	0.4 ± 1.4
Thigh circumference 15 cm above patella, cm	0.9 ± 1.8	0.9 ± 1.4	0.9 ± 1.4
Anteroposterior laxity, mm	−0.1 ± 1.6	1.5 ± 1.2^*b*^	1.1 ± 1.8
Pivot shift, mm/s^2^	0.6 ± 1.2	0.5 ± 0.8	0.3 ± 0.8

a Data are shown as mean ± SD. BPTB, bone–patellar tendon–bone; HT, hamstring tendon; HT + LET, hamstring tendon plus lateral extra-articular tenodesis.

b Statistically significant difference compared with the BPTB group (*P* = .049).

A total of 12 patients (20%) reported anterior knee pain: 5 (26%) in the BPTB group, 2 (10%) in the HT group, and 5 (23%) in the HT + LET group. Analysis did not reveal significant differences (*P* > .05).

The instrumental evaluation of knee laxity found no differences in the PS. At the same time, analysis showed a significant difference in AP laxity between the BPTB and HT groups (*P* = .049) (Table 4).

### Radiographic Outcomes

A final agreement in the evaluation of OA was reached for all 35 patients. A third evaluation by the senior orthopaedic surgeon was required for 4 of 35 patients for the medial compartment, 4 of 35 patients for the lateral compartment, and 5 of 35 patients for the patellofemoral joint.

**Figure 4.**
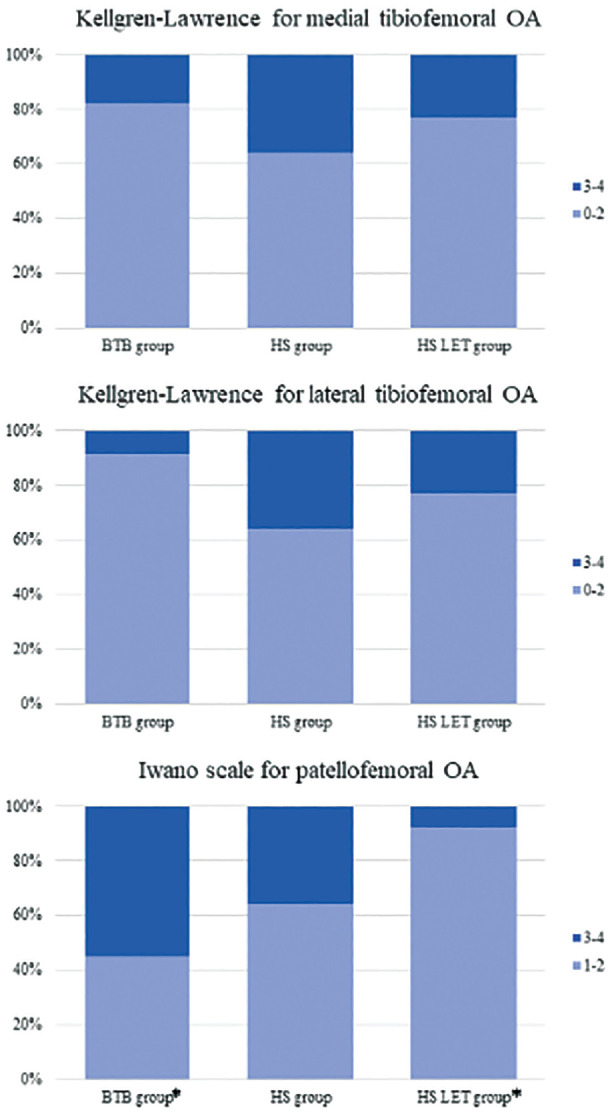
Graphical distribution of the patients according to the osteoarthritis (OA) grade. Note that the bone–patellar tendon–bone (BTB) group showed a greater prevalence of Iwano grades 3 to 4 (55% vs 36% and 8% for the hamstring tendon [HS] and hamstring tendon plus lateral extra-articular tenodesis [HS LET] groups, respectively), but the difference was statistically significant only between the BTB group and the HS LET group (asterisk).

The radiographic evaluation did not find statistically significant differences between the 3 groups when considering tibiofemoral medial and lateral compartments (Table 5 and Figure 4). On the other hand, the Iwano classification revealed a statistically significant difference between the HT + LET group and the BPTB group (*P* = .029) (Table 6), with the latter showing a higher prevalence of grade 3 to 4 patellofemoral OA (8% vs 55%, respectively) (Figure 4).

**Table 5:** Kellgren-Lawrence Grade for Tibiofemoral Osteoarthritis^*a*^

Grade	Medial Compartment			Lateral Compartment		
	BPTB Group (n = 11)	HT Group (n = 11)	HT + LET Group (n = 13)	BPTB Group (n = 11)	HT Group (n = 11)	HT + LET Group (n = 13)
0	2 (18)	0 (0)	3 (23)	2 (18)	0 (0)	2 (15)
1	4 (37)	5 (46)	4 (31)	6 (55)	6 (55)	4 (31)
2	3 (27)	2 (18)	3 (23)	2 (18)	1 (9)	4 (31)
3	2 (18)	2 (18)	2 (15)	1 (9)	2 (18)	3 (23)
4	0 (0)	2 (18)	1 (8)	0 (0)	2 (18)	0 (0)

a Data are shown as n (%). BPTB, bone–patellar tendon–bone; HT, hamstring tendon; HT + LET, hamstring tendon plus lateral extra-articular tenodesis.

**Table 6:** Iwano Grade for Patellofemoral Osteoarthritis^*a*^

Grade	BPTB Group (n = 11)	HT Group (n = 11)	HT + LET Group (n = 13)
1	2 (18)	3 (27)	10 (77)
2	3 (27)	4 (37)	2 (15)
3	6 (55)	1 (9)	1 (8)
4	0 (0)	3 (27)	0 (0)

a Data are shown as n (%). BPTB, bone–patellar tendon–bone; HT, hamstring tendon; HT + LET, hamstring tendon plus lateral extra-articular tenodesis.

### Subsequent Surgery and Failure

Among the 61 patients included in the final examination, 6 patients (10%) underwent revision surgery for graft failure: 3 (16%) in the BPTB group, 2 (10%) in the HT group, and 1 (5%) in the HT + LET group. Considering the additional criteria for clinical failure, 4 patients recorded a side-to-side difference in AP laxity >5 mm (1 patient in the BPTB group, 1 patient in the HT group, and 2 patients in the HT + LET group), and 6 patients showed a side-to-side difference in the PS >1.5 mm/s^2^ (3 patients in the BPTB group, 2 patients in the HT group, and 1 patient in the HT + LET group). Overall, the rates of clinical failure recorded were 37% for the BPTB group, 25% for the HT group, and 19% for the HT + LET group. Analysis did not reveal differences between the 3 groups for revision and clinical failure (*P* > .05).

Regarding subsequent surgery, in the BPTB group, 3 patients underwent medial meniscectomy in the index knee, and 2 patients underwent ACL reconstruction in the contralateral knee; in the HT group, 1 patient underwent medial meniscectomy in the index knee, and 4 patients underwent ACL reconstruction in the contralateral knee; and in the HT + LET group, 3 patients underwent medial meniscectomy in the index knee, 1 patient underwent ACL hardware removal, and 1 patient underwent ACL reconstruction in the contralateral knee. No statistically significant difference was found for subsequent contralateral ACL tears or meniscal injuries.

## Discussion

The main finding of the present study was that the 3 techniques provided comparably good clinical and radiographic outcomes at a mean follow-up time of 23 years. Additionally, the BPTB group demonstrated a slightly lower Tegner score than the HT + LET group, while the HT group showed greater AP laxity compared with the BPTB group in the long term.

The present prospective study began over 20 years ago to investigate differences between 2 of the most common ACL reconstruction techniques and compare them to a third one that included LET. To our knowledge, this study represents the first prospective and randomized clinical trial involving 3 different ACL reconstruction techniques with >20 years’ follow-up.

A greater incidence of OA is a common finding of studies investigating ACL reconstruction in the long term, with associated meniscectomy or chondral lesions identified as the main risk factors.^1,2,11,12,21,32,36^ On the other hand, there is no consensus regarding the impact of graft and surgical technique types on the long-term incidence of OA.^1,2,7,14,30,32^

Evaluating OA progression after ACL reconstruction is challenging because most studies were not comparative or did not have a sufficient follow-up time. Our study found no differences in the Kellgren-Lawrence grade between the 3 investigated techniques for the medial and lateral sides. The results were strengthened by patient selection, which excluded meniscal and chondral lesions at the time of surgery. Compared to studies with a similar follow-up time, Castoldi et al^2^ found a significant increase in the incidence of lateral OA at 19 years’ follow-up when LET was added to ACL reconstruction with the BPTB (59% vs 22%, respectively). However, they included patients who underwent lateral meniscectomy and did not analyze data based on the OA grade. Our results are consistent with previous case series with a minimum follow-up of 20 years, which reported an incidence of 17% to 27% of lateral high-grade OA in ACL reconstruction with LET.^21,36^

Furthermore, the study revealed a significant difference in the Iwano grade, with the BPTB group exhibiting a greater incidence of radiographic signs of patellofemoral cartilage degeneration than the HT + LET group. Castoldi et al^2^ compared operated knees (reconstruction with a BPTB with or without LET) to healthy contralateral knees and found a significant difference in patellofemoral OA between the 2 groups at 19 years, with the surgical knees showing an incidence of 67% versus 49% recorded in the healthy group. In their case series of knees undergoing ACL reconstruction with the BPTB with the modified Lemaire technique, Pernin et al^21^ recorded an incidence of 27% of radiographic IKDC grades C and D, not analyzing the patellofemoral compartment separately. Taking into account comparative studies, Holm et al^14^ did not find differences in radiographic OA between ACL reconstruction with a BPTB versus a 4-strand HT at 10 years. However, studies with a follow-up time similar to that in the present study revealed a greater incidence of high-grade OA for ACL reconstruction with the BPTB compared to the HT. Thompson et al^32^ did not separate the tibiofemoral and patellofemoral compartments for an evaluation; they found a higher incidence of radiographically detectable OA in the BPTB group compared to the HT group (61% vs 41%, respectively) at 20 years. This finding was strengthened by the exclusion of meniscal and chondral lesions in patient selection. Sajovic et al^26^ compared the BPTB with HT at 17 years and found an incidence of radiographic IKDC grades C and D in 33% of patients in the first group and 21% in the second one, with a statistically significant difference specifically for the patellofemoral and medial compartments. Therefore, a detrimental role of the BPTB graft on patellofemoral cartilage could be speculated, at least over a 20-year follow-up; in our cases, no statistically significant difference was found between the BPTB and HT groups likely because of the small sample size.

The clinical scores reported by our patients were globally good to excellent and consistent with those of previous studies with a similar follow-up time. In analyzing these scores, the mean Lysholm score for ACL reconstruction was between 86 and 94,^2,12,21,26,32,33,36^ and the mean subjective IKDC score ranged between 75 and 89 over a span of 17 to 24 years of follow-up.^2,12,21,26,27,32^ Comparative studies did not detect differences between various graft types^26,32^ or between ACL reconstruction with or without LET.^2^

One of the main findings was the slightly greater Tegner score in the HT + LET group compared to the BPTB group. The values for the HT and HT + LET groups were similar to those in previous studies, which reported scores between 2 and 7, with a median of 4.^12,33,36^ Castoldi et al^2^ did not evaluate the level of activity with the Tegner score but analyzed participation in pivoting sports; they found no difference between the BPTB and BPTB plus LET groups, with the first group at 67% participation in pivoting sports and the second at 69%. Similarly, long-term studies did not record differences in sports activity between ACL reconstruction with a BPTB and ACL reconstruction with an HT. Thompson et al^32^ found that 63% and 56% of patients who underwent ACL reconstruction with an HT and BPTB, respectively, participated in strenuous or very strenuous activities at 20 years. Sajovic et al^26^ recorded a Tegner score >6 in 29% of patients for ACL reconstruction with the HT and in 17% for ACL reconstruction with the BPTB at 17 years. Interestingly, although no statistically significant differences were found, a general trend could be identified in which ACL reconstruction with the HT seemed to produce greater activity levels in the long term.

Although both the BPTB and HT properly restored knee stability, the BPTB group showed a lower side-to-side difference in AP laxity compared to the HT group but not the HT + LET group. This finding is in line with those in the literature in the short term^3,9^; furthermore, the addition of LET was shown to influence not only the PS but also AP laxity.^5,13^ Interestingly, in studies with >10 years of follow-up, this objective laxity difference was often not reported.^14,26,32^

The revision and clinical failure rates recorded in the present study were 16% and 29%, respectively, for the BPTB group, 10% and 23%, respectively, for the HT group, and 5% and 19%, respectively, for the HT + LET group. Although a trend toward fewer revision procedures in the HT + LET group was noted, logistic factors (such as the long-term follow-up and the small number of participants available) limited sample size options. Therefore, the present data should be interpreted with caution, and future studies with larger cohorts may help us to understand if adding LET to ACL reconstruction is also protective at long-term follow-up. The data in the present study were, however, in line with those in studies with similar follow-up times.^2,21,27,32,33,36^ Moreover, comparative studies at 20 years detected no differences between ACL reconstruction with and without LET^2^ or between the HT and BPTB.^32^

### Limitations

The present study has several limitations. First, the size of the graft was not consistently reported in the surgical reports. Additionally, the contralateral leg was used as a reference for the objective laxity evaluation, even though some of the patients sustained knee trauma or minor surgery over the follow-up period. Similarly, OA incidence was only evaluated between the groups, and the contralateral healthy knee was not used as a comparison for this analysis. Moreover, the main limitations of the present study were the small sample size and the loss to follow-up for the clinical and radiographic examinations. Power analysis was performed to detect clinical differences; therefore, data regarding knee laxity and OA findings should be interpreted with caution. Additionally, there is no information regarding the possible reason for the slightly lower Tegner score found in the BPTB group (pain, instability, personal reason, etc). However, the strict inclusion criteria and study design (prospective and randomized controlled trial), combined with the long follow-up time and evaluation of 3 different ACL reconstruction techniques, made our study unique regarding ACL research.

## Conclusion

The 3 investigated techniques (BPTB, quadrupled HT, and over-the-top HT plus LET) provided comparable good clinical and radiographic outcomes at a mean follow-up time of 23 years. The BPTB group showed a greater prevalence of patellofemoral OA than the HT + LET group, while no difference was reported for tibiofemoral OA. The BPTB group revealed a slightly lower Tegner score than the HT + LET group, while the HT group showed slightly higher AP laxity than the BPTB group.
