# Corrigendum to ‘‘Long-term Outcomes After Anterior Cruciate Ligament Reconstruction With 3 Different Surgical Techniques: A Prospective Randomized Clinical and Radiographic Evaluation at a Minimum of 20 Years’ Follow-up’’

**DOI:** 10.1177/23259671251385668

**Published:** 2025-09-30

Lucidi GA, Agostinone P, Di Paolo S, Dal Fabbro G, Serra M, Viotto M, Grassi A, Zaffagnini S. Long-term outcomes after anterior cruciate ligament reconstruction with 3 different surgical techniques: a prospective randomized clinical and radio-graphic evaluation at a minimum of 20 years’ follow-up. *Orthop J Sports Med*. 2025;13(1): 23259671241302348.

This article has been updated to correct the first author affiliation to:

*Clinica Ortopedica e Traumatologica II, IRCCS Istituto Ortopedico Rizzoli, Bologna, Italy

